# Right Coronary Artery Pseudoaneurysm With Hemopericardium: A Rare Complication of Percutaneous Coronary Intervention

**DOI:** 10.7759/cureus.8567

**Published:** 2020-06-11

**Authors:** Sean Byrnes, Vijay Raj, Kunal Dhiren Gada

**Affiliations:** 1 Internal Medicine, State University of New York Upstate Medical University, Syracuse, USA; 2 Cardiology, State University of New York Upstate Medical University, Syracuse, USA; 3 Pulmonary and Critical Care, Stony Brook Medical University, Stony Brook, USA

**Keywords:** pci, rca, pseudoaneurysm

## Abstract

While percutaneous coronary intervention (PCI) is a commonly performed procedure, it still has many serious complications. Coronary artery pseudoaneurysms can form after PCI and can progress to cardiac tamponade. We report the case of an 80-year-old male who presented for an inferior wall ST elevation myocardial infarction, had drug-eluting stents placed to the right coronary artery (RCA), and subsequently suffered a RCA pseudoaneurysm with hemopericardium. He eventually underwent pseudoaneurysm repair with off pump coronary artery bypass graft. There is no established treatment protocol, and involvement of a multidisciplinary team improves outcomes.

## Introduction

The incidence of pre-existing coronary artery pseudoaneurysms is reported to be 5%, while the incidence of formation of a coronary artery pseudoaneurysm after drug-eluting stent (DES) placement is reported to be anywhere from 0.2% to 2.3% [1,2]. The mechanism by which coronary artery pseudoaneurysms form after DES placement is not well known, but theories include hypersensitivity vasculitis or direct wall damage. Complications of coronary artery pseudoaneurysms include rupture with subsequent cardiac tamponade. Treatment strategies vary widely, from conservative management to ligation of the aneurysm with bypass surgery.

## Case presentation

**Figure 1:**
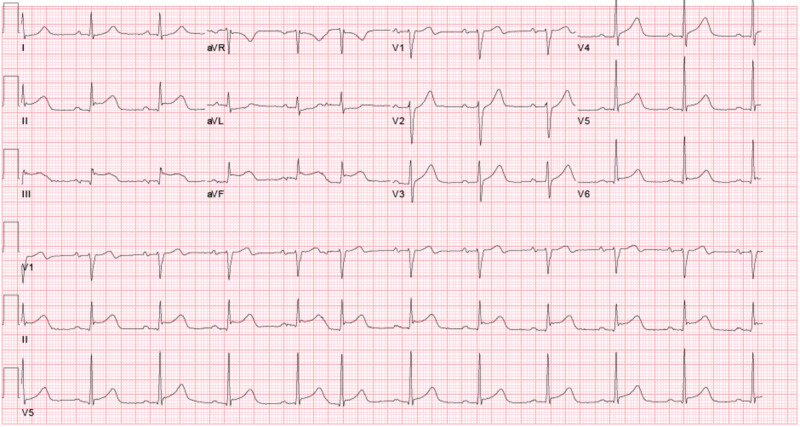
Electrocardiogram Note the ST segment elevations in leads II, III, and aVF.

An 80-year-old male with a past medical history significant for coronary artery disease, chronic obstructive pulmonary disease, hypertension, and type 2 diabetes mellitus presented with one day of dyspnea. On admission, he denied any chest pain, nausea, vomiting, palpitations, or weakness. Electrocardiogram (ECG) showed atrial flutter which did not improve with intravenous (IV) diltiazem, and therefore he was started on an IV amiodarone drip and IV heparin drip and admitted to the intensive care unit (ICU). His troponin I on admission was 0.02 ng/mL (reference < 0.04 ng/mL), and his ECG had no ischemic changes at that time. Later that night, he became bradycardic, his troponin I became elevated to 1.78 ng/mL, and a repeat ECG showed ST elevations in leads II, III, and aVF (Figure 1).

**Figure 2:**
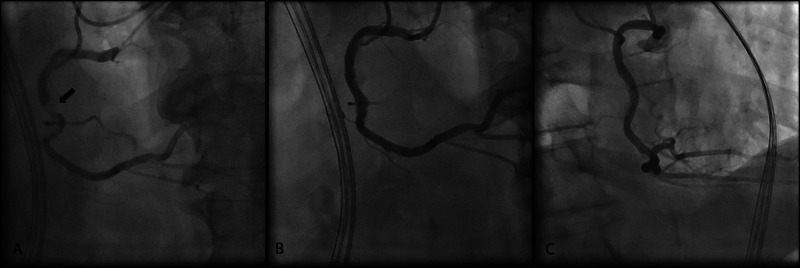
Coronary angiogram Coronary angiogram of the right coronary artery showing critical stenosis of the mid segment (arrow) on left anterior oblique view (A), which is widely patent without any thrombus, dissection, aneurysm, or perforation after percutaneous coronary intervention with drug-eluting stents as seen on left anterior oblique (B) and right anterior oblique (C) views.

The patient was subsequently taken for cardiac catheterization. Several lesions were seen in the mid right coronary artery (RCA). A 3.25 x 23 mm everolimus DES was deployed at the mid RCA lesion site and dilated to a maximum of 20 atmospheres (atm). Distal disease was noted so a 3.5 x 18 mm everolimus DES was deployed distal to the initial stent in an overlapping fashion and dilated to a maximum of 18 atm. The stents were post dilated to 22 atm. A 4.0 x 23 mm everolimus DES was then deployed at the proximal RCA lesion in overlapping fashion with the mid RCA stent and dilated to 16 atm and post dilated to 24 atm. A post injection image showed good stent position without any evidence of dissection, thrombus, or pseudoaneurysm (Figure 2).

**Figure 3:**
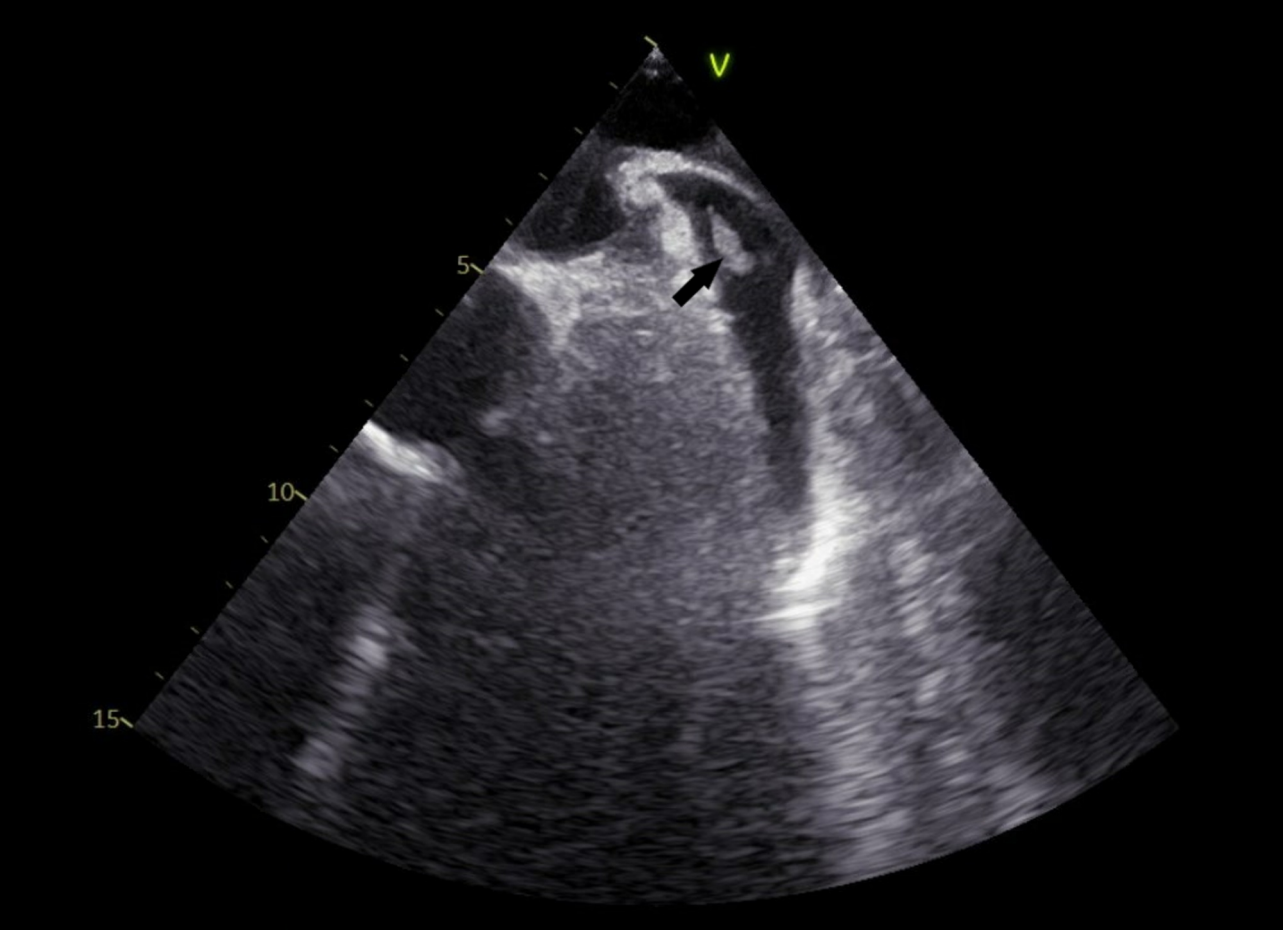
Transesophageal echocardiogram Left atrial appendage thrombus can be seen (arrow).

He was then started on oral antiplatelet therapy with aspirin and clopidogrel. After the cardiac catheterization, he was taken back to the ICU for close monitoring. He continued to be in atrial flutter so IV amidarone and IV heparin were continued. He also required IV metoprolol and IV diltiazem for rate control and due to this, he was planned for a transesophageal echocardiogram (TEE) and cardioversion. TEE done for the cardioversion showed a left atrial (LA) appendage thrombus, so the cardioversion was cancelled (Figure 3). Due to his LA appendage thrombus, he was then transitioned to apixaban and the patient subsequently converted to normal sinus rhythm the next day.

He was transferred out of the ICU and he was doing well until several days later when he experienced an episode of hemodynamically significant gastrointestinal (GI) bleed. An esophagogastroduodenoscopy (EGD) was performed which showed friable duodenal mucosa with active bleeding. Hemostasis was achieved with the application of hemospray. He also required five units of packed red blood cells (pRBC) and three units of fresh-frozen plasma (FFP) to improve his hematocrit, and due to this bleeding his anticoagulation was stopped. His hospital course was further complicated by methicillin-sensitive Staphylococcus aureus (MSSA) bacteremia for which he was treated with IV cefazolin.

**Figure 4:**
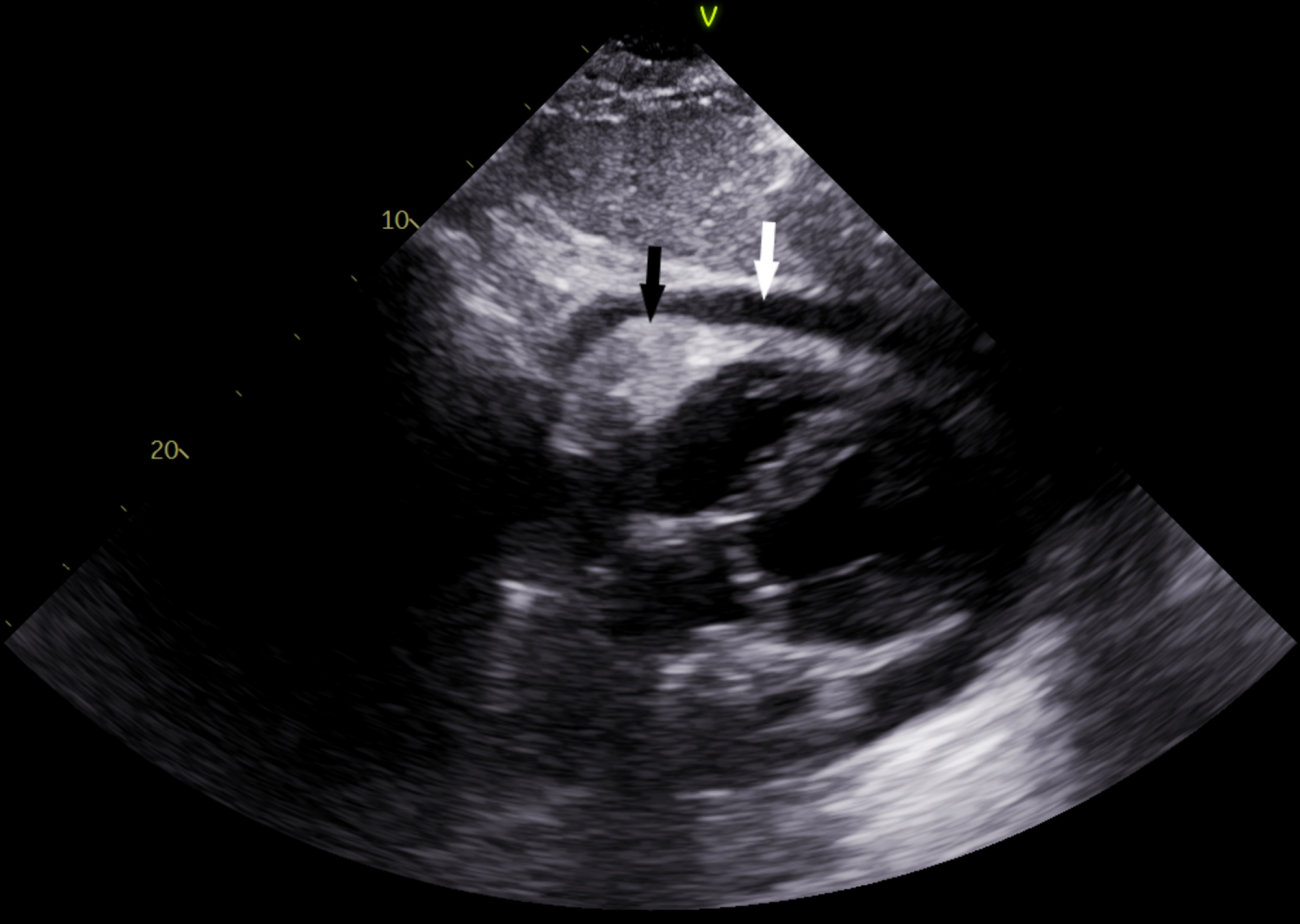
Transthoracic echocardiogram Pericardial effusion (white arrow) can be seen, along with organized component of the effusion (black arrow).

He was undergoing treatment when two weeks later he experienced sudden onset chest pain and shortness of breath. His blood pressure was 120/53 mmHg, heart rate was 122 beats per minute, respiratory rate was 22 breaths per minute, and oxygen saturation was 86% on room air. Due to his recent stent placement, a bedside transthoracic echocardiogram (TTE) was performed, which showed fibrinous material in the pericardium consistent with an organized hematoma without echocardiographic evidence of tamponade (Figure 4). Due to the fibrinous nature of the pericardial effusion seen on TTE, there was concern he would require a pericardial window.

**Figure 5:**
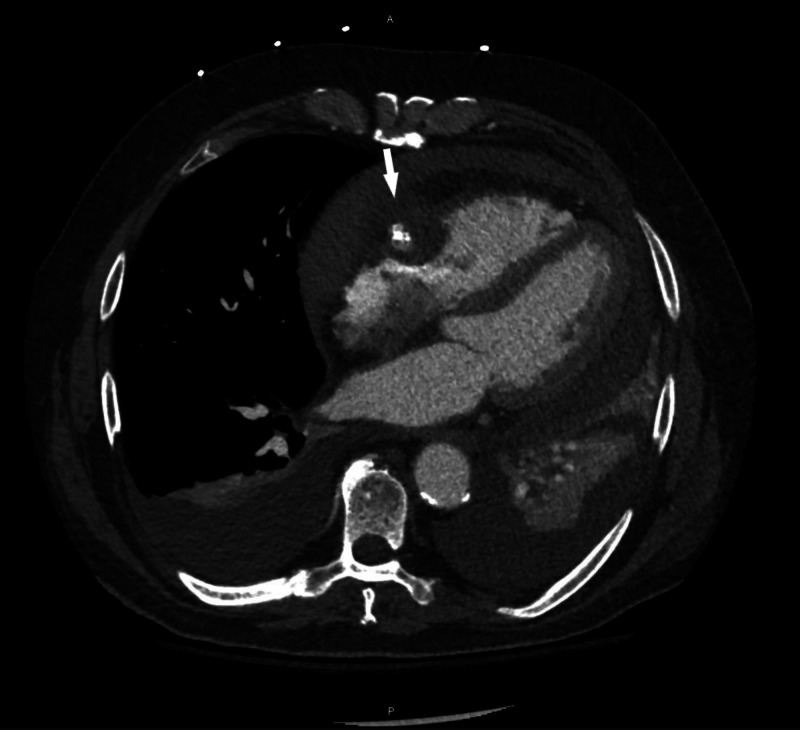
CT angiography of the thorax, axial view High-density pericardial effusion consistent with hemopericardium. High-density fluid can also be observed surrounding the right coronary artery, consistent with aneurysm (arrow). Bilateral pleural effusion can be seen.

**Figure 6:**
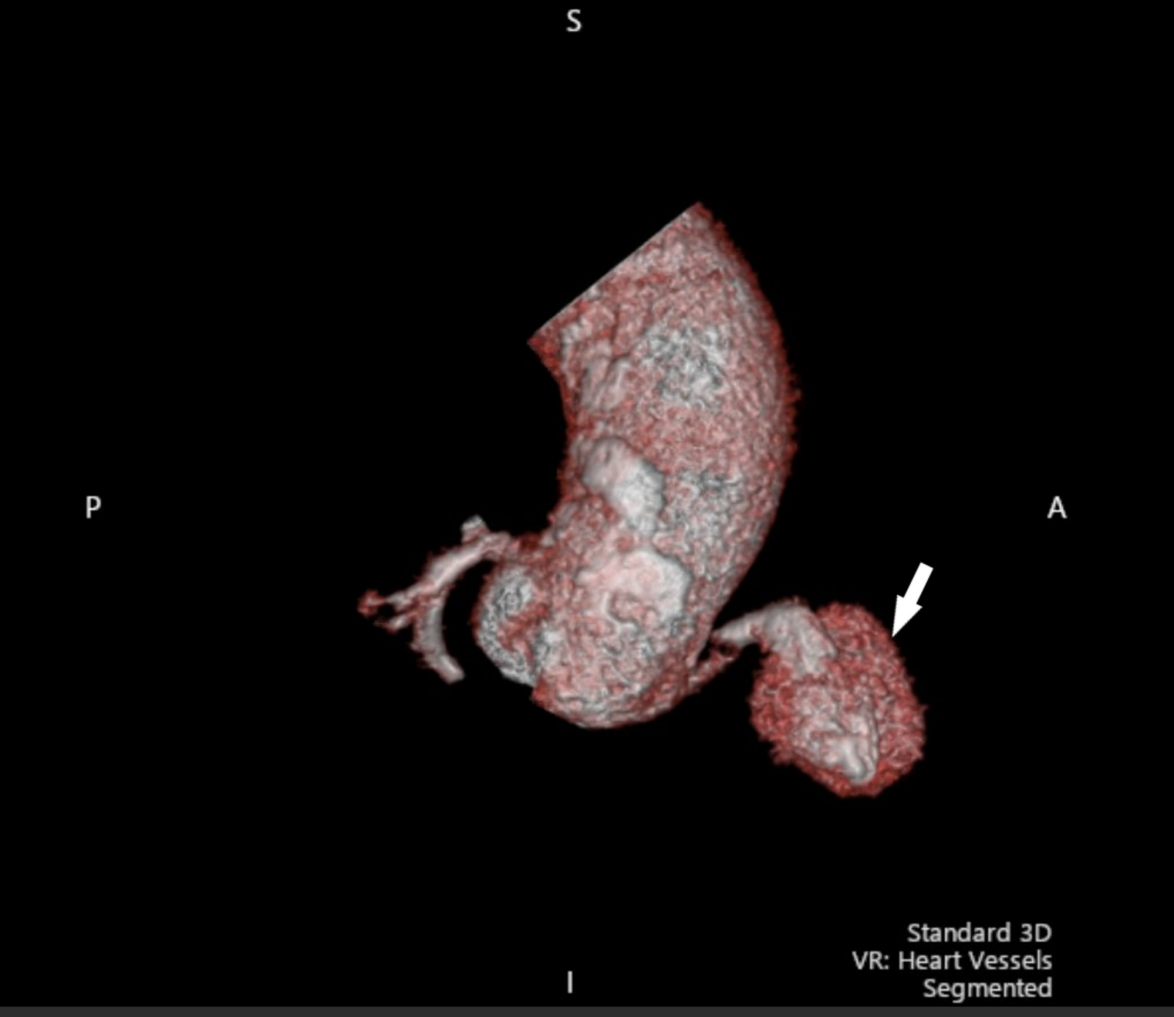
3-D reconstruction of the proximal ascending aorta Fluid can be seen surrounding the right coronary artery, consistent with pseudoaneurysm (arrow).

CT of the chest with contrast showed a pseudoaneurysm of the RCA in the right atrioventricular groove along with hemopericardium (Figures 5, 6). Bilateral pleural effusions were also seen.

After consultation with cardiothoracic surgery, the patient underwent off bypass single vessel coronary artery bypass graft (CABG) of the right internal mammary artery to the RCA along with decortication of the lung, heart, and repair of the RCA pseudoaneurysm. There was a layer of organized thrombus found in the pericardium without obvious fluid. The patient was transferred to the ICU after surgery and did well in the acute postoperative period.

In order to restart his anticoagulation, the patient had a follow-up EGD which again showed friable duodenal mucosa but no obvious bleeding. He was given a trial of IV enoxaparin sodium after which no signs or symptoms of any GI bleed were detected, and therefore he was restarted on apixaban. The next day, his hematocrit was noted to decrease requiring one unit of pRBC and so his anticoagulation and antiplatelet therapy were stopped. After the second episode of GI bleed, he remained in stable condition until he was discharged to a rehabilitation center.

## Discussion

The incidence of formation of coronary artery pseudoaneurysms after DES placement with or without subsequent tamponade is rare. Coronary artery pseudoaneurysms have been noted to develop anywhere from one week to four years post DES implantation [3]. The main associations with coronary artery pseudoaneurysms after DES placement have been oversized balloons, oversized stents, and atherectomy. The gold standard for diagnosis of a coronary artery pseudoaneurysm is by CT angiography. The pathophysiology of coronary artery pseudoaneurysm development is not well understood, but several theories exist as to the relationship between DES placement and pseudoaneurysm formation. One theory is related to the stent polymer. The stent apparatus is made up of the stent itself, the polymer, and the drug. It has been theorized that the polymer can cause a local hypersensitivity reaction which damages the wall, leading to formation of a pseudoaneurysm [4]. Other theories postulate that the specific drug on the DES leads to delayed healing with incomplete endothelization, which can lead to pseudoaneurysm formation [5]. As there have not been many studies addressing treatment, there are currently no guidelines. Treatment varies from medical management, such antiplatelets and/or anticoagulants, to surgical interventions, such as aneurysm ligation or resection [6].

Our patient experienced a coronary artery pseudoaneurysm with findings of hemopericardium; however, no active extravasation and tamponade physiology were seen. Our theory is that he developed a coronary artery pseudoaneurysm which ruptured and bled, and was made worse by the concurrent use of antiplatelet and anticoagulant therapy. The rupture then formed a clot before massive hemopericardium and cardiac tamponade could occur. Other causes of hemopericardium, including trauma or aortic dissection, are less likely as there was no reported trauma, and the CT did not show any aortic dissection [7]. We believe that the duration of time between the leak and his presentation was long enough that the blood vessel was able to form a clot, and therefore no active extravasation was seen. The exact reason for the pseudoaneurysm formation in our patient may not ever be known as the data showing the length of the lesion site are not available, and according to the operative report, the stents were not felt to be overdilated. Due to the coronary artery pseudoaneurysm and fibrinous pericardial effusion, our patient underwent surgical repair with CABG with good results. As there are no specific guidelines, early recognition and involvement of specialists along with individualized treatment is necessary to prevent life threatening complications.

## Conclusions

There are no specific guidelines for the treatment of RCA pseudoaneurysms; however, it has been suggested that there can be either conservative management or surgical management. This case illustrates how, due to the rarity of this entity, treatment must be individualized to the patient, involving a multidisciplinary team. In our patient, prompt surgical intervention prevented further life-threatening complications. In patients who recently underwent DES placement and who complain of chest pain, the clinician should have a high index of suspicion for procedural complications.

## References

[1] Chen D, Chang R, Ho A, Frivold G, Foster G (2008). Spontaneous resolution of coronary artery pseudoaneurysm consequent to percutaneous intervention with paclitaxel-eluting stent. Tex Heart Inst J.

[2] Kawsara A, Núñez Gil IJ, Alqahtani F, Moreland J, Rihal C, Alkhouli M (2018). Management of coronary artery aneurysms. JACC Cardiovasc Interv.

[3] Aoki J, Kirtane A, Leon MB, Dangas G (2008). Coronary artery aneurysms after drug-eluting stent implantation. JACC Cardiovasc Interv.

[4] Virmani R, Guagliumi G, Farb A (2004). Localized hypersensitivity and late coronary thrombosis secondary to a sirolimus-eluting stent. Circulation.

[5] Joner M, Finn A, Farb A (2006). Pathology of drug-eluting stents in humans: delayed healing and late thrombotic risk. J Am Coll Cardiol.

[6] Singh S, Goyal T, Sethi R (2013). Surgical treatment for coronary artery aneurysm: a single-centre experience. Interact Cardiovasc Thorac Surg.

[7] Levis JT, Delgado MC (2009). Hemopericardium and cardiac tamponade in a patient with an elevated international normalized ratio. West J Emerg Med.

